# Planned delayed retrieval of a migrated stent from an infected biloma via an endosonographically created duodenal route

**DOI:** 10.1055/a-2957-9722

**Published:** 2026-09-30

**Authors:** Shinji Oe, Yasuhisa Mori, Kenta Kajitani, Tsuyoshi Ueda, Nobuhiko Shinohara, Koichiro Miyagawa, Masaru Harada

**Affiliations:** 1 Third Department of Internal Medicine 13137 UOEHKitakyushuJapan; 2 Department of Surgery 1 13137 UOEHKitakyushuFukuoka PrefectureJapan

A 49-year-old woman with systemic lupus erythematosus and antiphospholipid syndrome was receiving high-dose corticosteroids and antithrombotic therapy. She underwent laparoscopic subtotal cholecystectomy for acute cholecystitis because of extensive collateral vessels associated with portal vein thrombosis. Two weeks later, a delayed bile leak with an infected biloma developed ( Fig. 1a, b ).

**Fig. 1:**
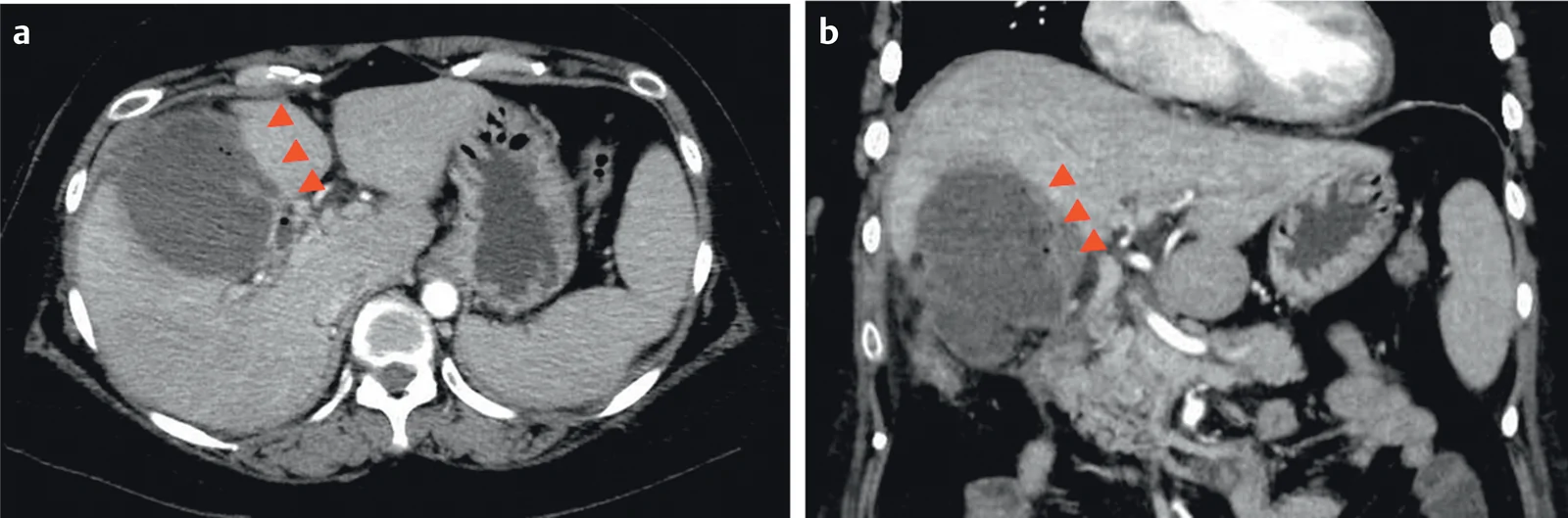
Contrast-enhanced computed tomography showing an infected postoperative biloma adjacent to the duodenum (arrowheads). ( **a** ) An axial image. ( **b** ) A coronal image.

Endoscopic ultrasound (EUS)-guided drainage is a minimally invasive option. 1​ Because repeat surgery was high risk, EUS-guided transduodenal drainage was performed. During the placement of a double-pigtail plastic stent, the stent migrated completely into the biloma cavity. Stent migration is a recognized adverse event. 2​ Immediate retrieval was deferred because infected bile impaired visualization. Two additional stents were placed, achieving infection control and biloma reduction ( Fig. 2a–c ).

**Fig. 2:**
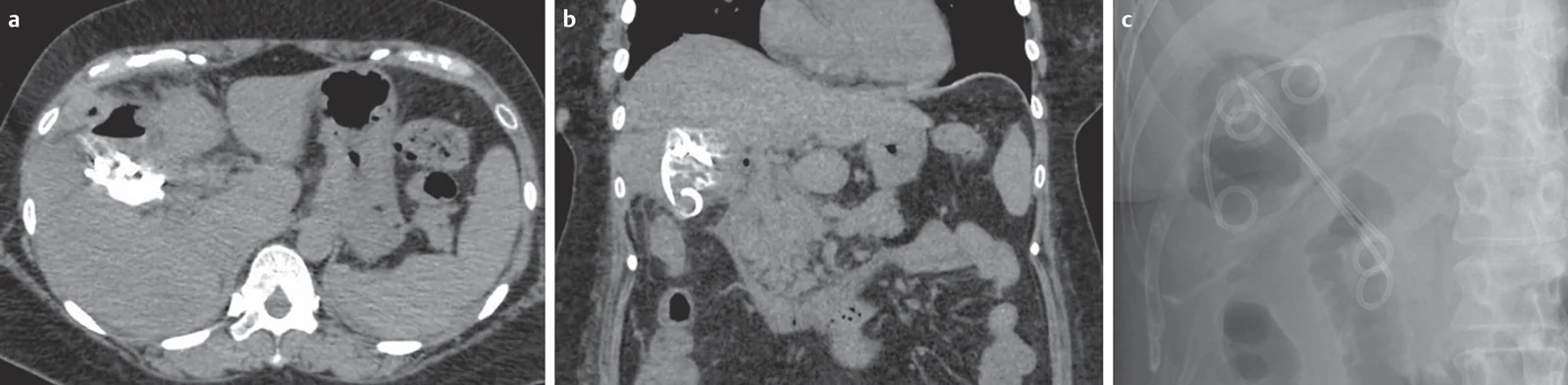
Imaging findings after migration of the double-pigtail plastic stent into the biloma cavity. ( **a** ) An axial computed tomographic image. ( **b** ) A coronal computed tomographic image. ( **c** ) An abdominal radiograph showing the migrated stent.

Five days later, the drainage stents were removed using a therapeutic endoscope (EG-840TP; Fujifilm, Tokyo, Japan), and the duodenal tract was dilated to 8 mm with a biliary dilation catheter (REN; Kaneka Medix, Osaka, Japan). An ultraslim endoscope (EG-6400N; Fujifilm; outer diameter, 5.9 mm) was advanced into the biloma cavity. The migrated stent was identified and retrieved under direct visualization using 1.8 mm grasping forceps (Fulton ATE; ASCH Japan, Tokyo, Japan; Video 1 ). Two double-pigtail plastic stents were placed for continued internal drainage ( Fig. 3a–c ). No food migration or related complications occurred. The patient recovered uneventfully and was discharged 7 days later. Given continued clinical improvement, routine follow-up computed tomography was not scheduled. Endoscopic removal of the indwelling stents was planned at 6 months.

**Video 1** Planned delayed endoscopic retrieval of a migrated double-pigtail plastic stent from an infected postoperative biloma through an endosonographically created duodenal tract using an ultraslim endoscope after infection control.


**Fig. 3:**
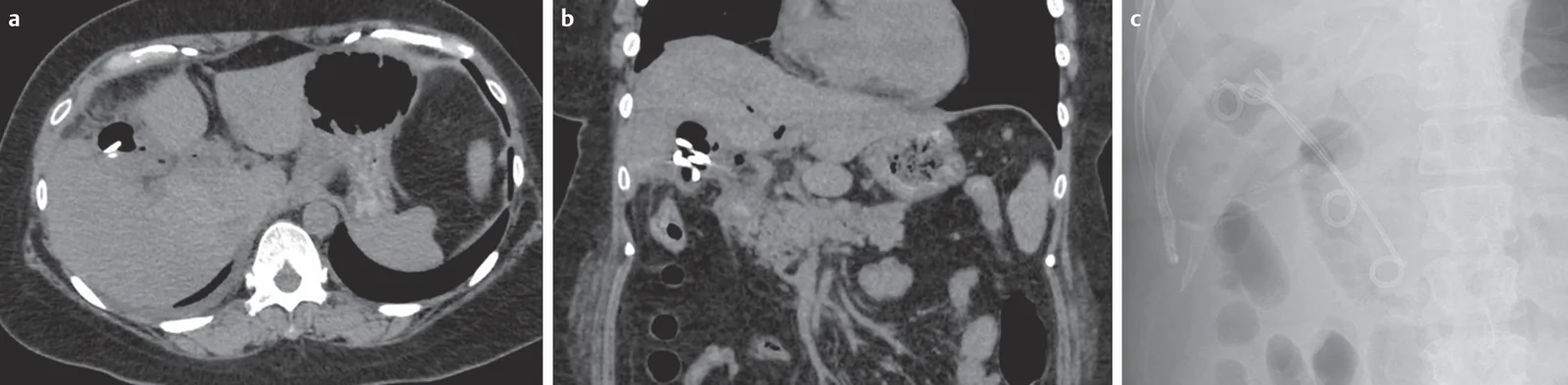
Imaging findings after the endoscopic retrieval of the migrated stent. ( **a** ) An axial computed tomographic image. ( **b** ) A coronal computed tomographic image. ( **c** ) An abdominal radiograph confirming the successful retrieval of the migrated stent and placement of two double-pigtail plastic stents.

Endoscopic retrieval of migrated stents from abscess cavities has been reported. 3​ 4​ ​5​ However, the planned delayed retrieval from an infected postoperative biloma through an endosonographically created duodenal route has not been reported. This approach may provide a minimally invasive salvage option. Although the optimal timing remains uncertain, a short delay after infection control may allow tract stabilization while avoiding granulation-related stent embedment.

Endoscopy_UCTN_Code_CPL_1AM_2AZ

## Declaration of GenAI Use

The authors confirm that no generative artificial intelligence was used in the preparation of this manuscript.
